# *Cox-2 *gene overexpression in ureteral stump urothelial carcinoma following nephrectomy for renal cell carcinoma: a case report

**DOI:** 10.1186/1752-1947-6-44

**Published:** 2012-01-30

**Authors:** Wei-Pin Chang, Tsu-Ming Chien, Yu-Shiuan Wang, Siou-Jin Chiu, Mei-Hui Lee, Wei-Chiao Chang, Yii-Her Chou, Ming-Feng Hou

**Affiliations:** 1 Department of Healthcare Management, Yuanpei University, HsinChu, Taiwan; 2 School of Post-baccalaureate Medicine, Kaohsiung Medical University, Taiwan; 3 Department of Medical Genetics, Kaohsiung Medical University, Taiwan; 4 Department of Urology, Kaohsiung Medical University Hospital, Taiwan; 5 Cancer center, Kaohsiung Medical University Hospital, Kaohsiung, Taiwan; 6 Department of Urology, Kaohsiung Medical University, Taiwan

## Abstract

**Introduction:**

A primary ureteral stump tumor after a nephrectomy is rare; urothelial carcinoma of the ureteral stump after a nephrectomy for renal cell carcinoma is even rarer. A thorough review of the literature indicated that only seven cases have previously been reported. In this study, we report the first Taiwanese case of urothelial carcinoma of the ureteral stump after a nephrectomy. It is also the first female case in the literature. The relationship between inflammatory genes, medication history and ureteral stump carcinoma after a nephrectomy for renal cell carcinoma has not been reported.

**Case presentation:**

A 72-year-old Asian Taiwanese women with chronic hepatitis C, liver cirrhosis and chronic kidney disease underwent a hand-assisted laparoscopic radical nephrectomy in 2001 due to renal cell carcinoma. Nine years later, she was diagnosed with ureteral stump urothelial carcinoma. Genetic and medication surveys were performed. Importantly, our patient had taken Chinese herbal drugs for more than 10 years and the inflammatory gene, *Cox-2*, was highly expressed in this patient. This is the first report to study the relationship between the *Cox-2 *gene and ureteral stump carcinoma after a nephrectomy for renal cell carcinoma.

**Conclusion:**

Long-term multiple use of Chinese herbal drugs could be one of the important risk factors for developing urothelial cancer. Close functional coupling between Chinese herbal drugs, *Cox*-2 gene activation and urothelial cancer should be further investigated.

## Introduction

The incidence of urothelial carcinoma in the ureter is rare, accounting for less than 5% of all urothelial neoplasms. It often occurs in the lower third of the ureter and the recurrence rate is relatively high [1]. A primary tumor of the ureteral stump after a nephrectomy is infrequently observed. Moreover, a primary tumor of the ureteral stump after a nephrectomy for a renal cell carcinoma (RCC) is extremely rare. A thorough review of the literature indicated that only seven cases have previously been reported of ureteral stump carcinoma after a nephrectomy for an RCC [2-8]. This eighth observed case is the first reported case in Taiwan.

Prostaglandins are important inflammatory molecules involved in the pathogenesis of cancer. Prostaglandins can be metabolized from arachidonic acid by the cyclooxygenases 1 (Cox-1) and cyclooxygenases 2 (Cox-2). Cox-1 is a constitutive enzyme whereas Cox-2 is an inducible enzyme that is activated by extracellular stimulations including cytokines [9], growth factors [10] and endotoxins [11]. Several lines of evidence indicate that Cox-2 is one of the most critical enzymes in tumor metastasis. Cox-2 overexpression may lead to the cancer cell invasiveness of human breast cancer cells [12]. Clinical studies have extensively documented that Cox-2 inhibitors, such as nonsteroidal anti-inflammatory drugs, can reduce the incidence of colon cancer [13].

To the best of our knowledge, the relationship between inflammatory genes, medication history and ureteral stump carcinoma after a nephrectomy for an RCC has not been reported. We therefore conducted a prospective study to identify the potential risk factor in the first reported case of ureteral stump carcinoma after a nephrectomy for an RCC in Taiwan.

## Case presentation

**Figure 1:**
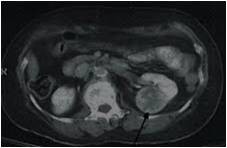
**Axial view of contrast-enhanced computed tomography scans revealing a left renal tumor**.

A 72-year-old Asian Taiwanese woman with intermittent, painless, total gross hematuria visited the Department of Urology of our hospital in 2010. She had been diagnosed with chronic hepatitis C and stage III chronic kidney disease for 10 years and has regular follow-up. In 2001 (nine years ago), a routine ultrasound examination showed a left renal tumor. Computed tomography (CT) confirmed the diagnosis (Figure 1). She then was transferred to our Department of Urology and underwent a hand-assisted laparoscopic radical nephrectomy. The pathological reports revealed an RCC of stage T1N1M0, which covered the cortex and medulla with central areas of necrosis and hemorrhage, measuring 11.0 cm × 7.0 cm × 4.0 cm. The calyx segment of the collecting system was also included. Her ureter was not dilated and its mucosa was not remarkable. Fortunately, her ureter was free from cancer tissues. After the operation, she received regular follow-up.

**Figure 2:**
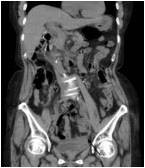
**Coronal view of computed tomography scans revealing a prominent soft tissue at the left ureterovesical junction**. The middle and lower third parts of her residual ureter were dilated.

At the beginning of 2010, nine years later, our patient had one episode of gross hematuria. The results of examinations such as urine cytology and cystoscopy were normal. A CT scan showed two renal stones in her right kidney without other abnormalities. The hematuria subsided spontaneously. Two months later, another episode of gross hematuria occurred. This time, clot formation was noted in her urine. Once again, the symptoms subsided spontaneously. Cystoscopy revealed whitish debris coating the left ureter orifice; no gross bladder tumor was seen. A ureter catheter could not be inserted into her left ureter due to a ureterovesical junction stricture. Thus, retrograde pyelography with a cone tip ureteral catheter was tried, but also failed. However, CT scans showed a prominent soft tissue mass at the ureterovesical junction. The middle and lower third parts of her residual left ureter were dilated (Figure 2). According to the imaging, a urothelial carcinoma of the ureter stump was highly suspected. Therefore, a ureteroscopic examination and biopsy was performed using a fiberoptic rigid ureteroscope. We tried to use a guidewire to pass the ureteral orifice cystoscopically but the guidewire could only be progressed for less than 1 cm. The ureteroscope was therefore inserted without intramural dilatation of the ureterovesical junction and it was only possible to examine the ureter up to less than 1 cm from the ureterovesical junction. The biopsy was thus taken from the lower ureter where it is close to the ureterovesical junction. The pathological report showed a high-grade infiltrating urothelial carcinoma. The tumor invaded the subepithelial connective tissue (lamina propria) and the pathologic staging of the primary tumor was T1. A radical operation (ureterectomy with ipsilateral bladder cuff excision or a cystectomy) or systemic chemotherapy were suggested. Without family support, our patient had been in a depressed state for a long time. With consideration of the multiple coexisting systemic diseases, such as liver cirrhosis, chronic kidney disease, gallbladder stones, facial palsy, hypertension and recurrent urinary tract infection, our patient refused both treatment options. She asked for supportive treatment only.

**Figure 3:**
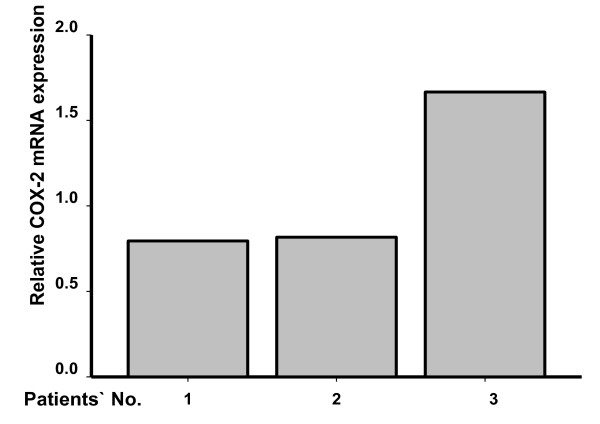
**The *Cox-2 *gene expression level was checked in patients with calcium nephrolithiasis (patients 1 and 2) or with a urothelial carcinoma of the ureteral stump (patient 3)**.

To further identify the possible risk factors contributing to the urothelial carcinoma of the ureteral stump, we checked the medication history of our patient. As shown in Table 1, our patient had taken several different kinds of drugs for more than 10 years. These were cephalosporin, nitroxoline, sulfonamide, levofloxacin for urinary tract infection, atenolol, amlodipine, solantin for hypertension, atorvastatin for hyperlipidemia, ursodeoxycholic acid for liver cirrhosis and Chinese herbal drugs for multiple somatic complaints. Importantly, although our patient had taken Chinese herbal drugs for more than 10 years, the compositions of these drugs remain unclear. Long-term multiple use of Chinese herbal drugs may influence inflammatory reactions. We therefore compared gene expression level of *Cox-2 *between this patient and patients with calcium nephrolithiasis. Real-time polymerase chain reaction experiments indicated that a high level of expression of the *Cox-2 *gene was seen in our patient with the urothelial carcinoma of the ureteral stump (Figure 3).

**Table 1:** Medication history and the possible side effects

Drug	Disease/disturbance	Side effect
Cefradine	Urinary tract infection	Hypersensitivity, elevated liver enzyme, diarrhea
Nitroxoline	Urinary tract infection	Gastrointestinal intolerance, urine coloration
Sulfonamide	Urinary tract infection	Hypersensitivity, folic acid deficiency, hematological abnormality
Levofloxacin	Urinary tract infection	Diarrhea, tendinitis, seizure neuropathy, elevated liver enzyme
Atenolol	Hypertension	Bradycardia, cough, Raynaud's syndrome
Amlodipine	Hypertension	Flush, tachycardia, edema
Solantin	Thromboembolism	Dizziness, abdomen distress, angina, headache
Ursodeoxycholic acid	Liver cirrhosis	Gastrointestinal disturbance
Atorvastatin	Hyperlipidemia	Gastrointestinal disturbance, elevated liver enzyme, myopathy, neuropathy
Chinese herb drugs	Multiple somatic complaints	Unknown

## Discussion

An RCC is a common renal parenchymal malignant tumor. RCCs originate from the proximal renal tubular epithelium in the renal cortex. Urothelial carcinoma is a common cancer of the urinary bladder. It may also occur in the collecting system of the upper urinary tract, such as the renal pelvis and ureter. The causes of RCCs are unknown; environmental exposure to asbestos, solvents and cadmium, smoking and genetic mutation have been implicated. The causes of urothelial cancer include smoking and occupational exposure to chemicals, dyes, rubbers, petroleum, leathers and printing materials; people who are exposed to these for a long time are a high-risk group for developing urothelial cancers. Genetic changes may also occur throughout the urothelium.

A primary ureteral stump tumor can be defined as a tumor occurring in the closed ureteral stump after a nephrectomy for either benign or malignant disease other than a urothelial tumor. Clinically, both diagnoses and treatments for a ureteral stump tumor are still elusive. Furthermore, a primary urothelial carcinoma of the ureteral stump after a nephrectomy for an RCC is extremely rare. Only seven cases were reported in the literature (Table 2). Our case is the first case in Taiwan and is the first female case in the literature.

**Table 2:** Patient data for a ureteral stump tumor after a nephrectomy for a renal cell carcinoma

Case	Age	Sex	Interval (years)	Side	Reference
1	75	M	Two and a half	Right	[4]
2	62	M	Two and a half	Right	[5]
3	70	M	Seven	Right	[3]
4	49	M	23	Left	[2]
5	88	M	Six	Left	[6]
6	64	M	Six	Right	[8]
7	68	M	0.9	Right	[7]
8	72	F	Nine	Left	Present case

In the reported cases, the mean interval between the nephrectomy and detection of the ureteral stump tumor was 7.1 ± 7.0 years, ranging from 0.9 years to 23 years. In a report by Kim *et al*., the interval between the detection of a ureteral stump tumor and the nephrectomy for benign renal disease was 76.5 months [14]. Although the etiology of a ureteral stump tumor remains to be seen, metaplastic changes caused by chronic inflammation due to infection or stone irritation, malignant metamorphosis caused by leukoplakia [15], exposure to carcinogenic substances, a high risk of genetic cancer susceptibility and several others factors are very likely to be associated with this rare disease. Chronic inflammation is often related to squamous cell metaplasia resulting in squamous cell carcinoma [16]. After a nephrectomy, the ureteral stump is no longer exposed to the chemical carcinogens which are present in the urine; resulting in a low possibility of developing cancer from a urinary carcinogen. Genetic hypersensitivity, mutation or pharmacological carcinogenetic effects provide other possibilities for cancer development. Our patient had experienced chronic recurrent urinary tract infection, with episodes occurring three to four times every year. Several reports have indicated the correlation between Chinese herbal drugs and urothelial carcinoma [17-20]. A patient with liver cirrhosis, such as ours, may have a reduced ability to metabolize herbal drugs due to damage to the liver. Therefore, the side effects of Chinese herbal drugs may be amplified and accumulate.

In addition to chemical carcinogenesis from Chinese herbal drugs, genetic effects might contribute to malignant tumors. *Cox-2 *polymorphism has been shown to be a predictive marker of survival in non-small cell lung cancer patients treated with chemoradiotherapy or radiotherapy [19,21,22]. Our results, consistent with previous reports, indicated that a high level of *Cox-2 *gene expression may be associated with the pathogenesis of the primary ureteral stump tumor. The *Cox-2 *gene can be evoked by a variety of chemical compounds, including alkaloids, polysaccharides and heavy metals. Chinese herbal drugs contain a large amount of alkaloids, polysaccharides and heavy metals. Long-term use of Chinese herbal drugs may result in physiological dysfunction. Another postulation by Klee and colleagues [5] was that a prior bladder carcinoma could make reflux implantation into the ureteral stump more likely, even though no significant vesicoureteral reflux was noted. In other words, a history of previous cancer elsewhere in the urinary tract was thought to be a high risk factor for ureteral stump cancer. Unlike the assumption proposed by Klee and colleges, the patient in our case had no history of bladder cancer, but her ureteral stump had possibly been exposed to urinary carcinogens for more than 10 years because of vesicoureteral reflux.

## Conclusions

Urothelial carcinoma of the ureteral stump after a nephrectomy for an RCC is rare. A ureteral excision made during a nephrectomy should be as large as possible for benign disease or a non-urothelial tumor. If the ureter is left after the nephrectomy, the ureteral stump should be evaluated and followed-up carefully. Long-term multiple use of Chinese herbal drugs could be one of the important risk factors for developing urothelial cancer. Close functional coupling between Chinese herbal drugs, *Cox-2 *gene activation and urothelial cancer should be further investigated.

## Consent

Oral and written informed consent was obtained from the patient and her next-of-kin for publication of this case report and any accompanying images. A copy of the written consent is available for review by the Editor-in-Chief of this journal.

## Competing interests

The authors declare that they have no competing interests.

## Authors' contributions

WPC drafted the article. WPC, TMC and YSW analyzed and interpreted the patient data. MHL photographed and interpreted the pathologic findings. WCC, MFH, WPC and SJC took part in the critical revision and YHC took part in the diagnosis, treatment of the patient and gave final approval of the manuscript. All authors have made substantive intellectual contributions to this study and to the manuscript and have read and approved the final manuscript.
